# The Role of γ-Polyglutamic Acid, Superphosphate, and Smectite-Silica Clay Additives in Stabilizing Organic Matter, Reducing the Bioavailability of Heavy Metals, and Limiting the Ecotoxicity of Composts Made from Poultry Litter and Biochar

**DOI:** 10.3390/ma19091788

**Published:** 2026-04-28

**Authors:** Krzysztof Gondek, Agnieszka Baran, Michał Kopeć, Piotr Micek, Iwona Spałek

**Affiliations:** 1 Department of Agricultural and Environmental Chemistry, University of Agriculture in Krakow, Al. Mickiewicza 21, 31-120 Krakow, Poland; krzysztof.gondek@urk.edu.pl (K.G.); michal.kopec@urk.edu.pl (M.K.); iwona.spalek@urk.edu.pl (I.S.); 2 Department of Nutrition, Animal Biotechnology and Fisheries, University of Agriculture in Krakow, Al. Mickiewicza 24/28, 30-059 Krakow, Poland; piotr.micek@urk.edu.pl

**Keywords:** composts, polyglutamic acid, superphosphate, smectite-silica clay, humic compounds, metal mobility, ecotoxicity

## Abstract

A mixture of poultry litter (PL) and biochar (BC) was composted over 120 days in a bioreactor. To assess the impact on the stability of organic matter, the bioavailability of heavy metals, and ecotoxicity, the PL+BC biomass was supplemented with 0.5% (*w*/*w*) γ-polyglutamic acid (PGA), superphosphate (SPP) and smectite-silica clay (SSC) relative to the dry matter. Incorporating PGA, SPP, and SSC additives into PL+BC increased total carbon content by an average of 6%, compared to PL+BC without additives. The SSC additive proved to be more effective in increasing the humic acid carbon content, raising Cha by an average of 23% relative to PGA and SPP treatments. The incorporation of biochar into PL led to a substantial increase in nonhydrolizing carbon content, while the enrichment of composts with PGA, SPP, or SSC resulted in an escalation in this form of carbon by an average of over 7% compared to PL+BC. The lowest amounts of metals extracted with water and the lowest RAC values were obtained for PL+BC+SPP compost. The additives used stabilized the composts more quickly and reduced their toxicity. The classification of PL compost was designated as class III, whereas composts that incorporated additives were classified as class II toxicity. The study findings substantiated the necessity to incorporate additives during the biological processing of poultry litter.

## 1. Introduction

According to United Nations projections, the world’s population is set to rise [1]. A larger population invariably leads to heightened demand for critical resources such as energy, housing, transportation, and most crucially, water and food. An escalating global consumption has a destructive impact on the Earth’s resources and may increase the risk of large-scale disasters such as pandemics or environmental degradation. As the world population grows and consumption levels rise, the amount of waste generated will also increase. This includes waste generated during food production cycles.

Poultry farming is a highly effective method of animal husbandry that ensures food security for a substantial part of the global population [2,3]. In 2024, the global production of poultry meat reached 103.7 million tons, and forecasts for 2025 estimate an increase to 105.8 million tons. However, such intensive poultry farming leads to a significant environmental footprint. This phenomenon is primarily attributable to the release of pollutants (including greenhouse gases) and the disposal of waste materials, such as manure [3]. Improper management of the breeding process, particularly the improper disposal of waste materials following completed breeding cycles, can pose a serious threat to the environment and human health. According to Devendran Monogaran et al. [2], a chicken produces 80 g to 100 g of manure daily, corresponding to 3–4% of its body weight. On a global scale, this is a huge amount of manure, the use of which in its unprocessed form can have adverse consequences for the environment.

Poultry litter may contain pesticide residues, pathogens, drugs (antibiotics), hormones, heavy metals, macronutrients (in inappropriate proportions), and other contaminants that can lead to deterioration in air, soil, and water quality, as well as the emergence of antimicrobial-resistant pathogen strains. It should also be noted that the accumulation of this type of material is not environmentally neutral due to the biochemical processes that result in the release of gaseous substances [4].

Among the methods used to transform poultry litter, composting is a process that yields a safe product that improves soil fertility and is beneficial for the reclamation of degraded land and the revitalization of post-industrial areas [5,6]. Compost can also be a component of substrates used in plant cultivation and acts as a fungistatic agent in the soil environment [7,8].

The fundamental prerequisite for the effective execution of the composting process is the judicious selection of components (in terms of C/N ratio) and the possibility of adequate gas exchange (aeration). These are the main factors determining composition of the microorganism community, which shape their ability to degrade biomass [9]. The water content and pH value of the biomass are also important for the efficiency of the composting process. According to Sokač et al. [10], all these variables and their relationships must be considered simultaneously to determine the operational efficiency of the composting process. Poultry litter is characterised by low porosity, a high moisture content, a narrow C/N ratio and a high pH. The incorporation of suitable additives such as rice husks, wood chips, or biochar effectively improves the properties of poultry litter intended for composting. These additives increase the C/N ratio, improve the porosity of poultry litter, and increase aeration, while reducing the water content. Zhang et al. [11] found that combining poultry litter, tobacco residues, mushroom bran and biochar in the composting process is environmentally friendly and economically viable. From an environmental perspective, the impact of composted materials on the soil, including the population of microorganisms and plants, is of great importance.

In recent years, a variety of pragmatic methodologies have been implemented to enhance the composting process and reduce its negative environmental impact, such as adjusting the physicochemical parameters of biomass [10,12], changing aeration parameters [13], and using various types of additives [14,15]. Of all the additives, biochar and zeolites are the most widely used to improve the transformation of organic matter and reduce nitrogen losses, among other functions [15,16]. Chen et al. [16] and Awasthi et al. [15] demonstrated that the addition of biochar facilitates the degradation of organic matter during sewage sludge composting. Dias et al. [12] and Jindo et al. [17] showed that the simultaneous composting of manure and biochar can accelerate the humification of organic matter and improve compost quality. Conversely, Zhang and Sun [18] found that adding zeolite significantly improves the decomposition of lignocellulose and increases the humic acid content during plant waste composting. Hao et al. [19] found that adding biochar and montmorillonite to composted poultry litter substantially decreased the mobility of copper and zinc. At the same time, these additives significantly altered the structure of the bacterial community and strengthened the correlation between bacterial groups and heavy metal fractions. According to Chen et al. [20], the incorporation of superphosphate into composted materials on an industrial scale has the potential to reduce greenhouse gas emissions and enrich the soil with nutrients. Although the economic benefits of adding superphosphate to reduce greenhouse gas emissions are limited, this technology has considerable economic potential beyond the scope of nitrogen savings. Adding 18% superphosphate was associated with a lower ecological risk, yet it still increased the availability of Cu, Mn and Fe in pig manure compost compared to the control group [21]. The addition of superphosphate to the compost reduced the number of bacteria and increased the diversity of the fungal population compared to the control. Redundancy analysis revealed a significant correlation between heavy metal fractions and the composition of bacteria and fungi in composted pig manure with the addition of 18% superphosphate [21].

Considering the legal and environmental restrictions on poultry production, this study involved the composting of a mixture of poultry litter (PL) and biochar (BC). In order to assess changes in organic matter stability, heavy metal bioavailability, and ecotoxicity of composts, γ-polyglutamic acid (PGA), superphosphate (SPP), and smectite-silica clay (SSC) were used as additives. The objective of the experiment was to: (i) examine the effect of additives on the content and fractions of humic compounds, (ii) assess changes in the bioavailability of selected heavy metals, and (iii) evaluate alterations in the ecotoxicity of the composts produced.

## 2. Materials and Methods

### 2.1. Materials and Composting Processes

The study was conducted under laboratory conditions on composts prepared from poultry litter (PL) and biochar (BC). The proportion of components in the composting biomass was 50% PL and 50% BC. The composted biomass was supplemented with γ-polyglutamic acid (PGA), superphosphate (SPP), and smectite-silica clay (SSC). The addition of SSP, SSC, and PGA was 0.5% (relative to the dry matter of the mixture). The same dose (0.5%) was applied for all additives to enable direct comparison of their effects under uniform conditions. This dose was selected as a representative low-level application based on preliminary studies.

The poultry litter (PL) was derived from domestic guinea fowl breeding, and the biochar (BC) was produced from coniferous tree waste at 450 °C [22]. The study also utilized chemically pure γ-polyglutamic acid (PGA) (commercial product, LonierHerb, Xi'an, China), superphosphate (SPP) (commercial product, Siarkopol Tarnobrzeg sp. z o.o., Tarnobrzeg, Poland), and smectite-silica clay (SSC) obtained from the Dylągówka–Zapady deposit in south-eastern Poland [23]. The chemical parameters of the materials can be found in Table 1.

**Figure 1:**
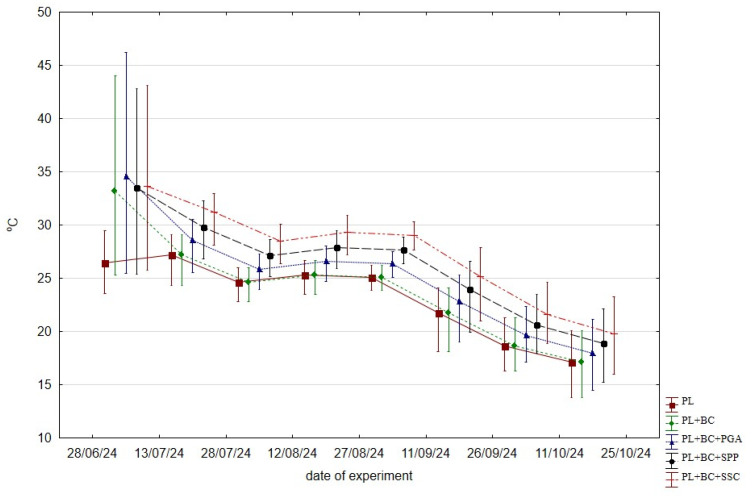
Average temperature (±SD) of composts in 15-day periods during the experiment.

Composting was carried out over 120 days, from 28 June to 25 October 2024. It was conducted in a 1.0 × 0.9 × 0.8 m bioreactor containing 11 kg of fresh composted material in perforated containers. The bioreactor chamber was isolated from external conditions and aerated 6 times a day at a rate of 0.015 m^3^ min^−1^. To improve aeration and homogenization, the composted biomass was manually mixed once a week. A Steinberg^®^ recorder was used to record the temperature of the biomass during composting. The measurement results are shown in Figure 1.

The experimental design included the following treatments: PL—poultry litter without additives, PL+BC—poultry litter + biochar, PL+BC+PGA—poultry litter + biochar + γ-polyglutamic acid, PL+BC+SPP—poultry litter + biochar + superphosphate, PL+BC+SSC—poultry litter + biochar + smectite-silica clay.

### 2.2. Chemical Analyses of Materials and Composts

The pH and electrical conductivity (EC) were measured in the samples (material:water = 1:5) using a pH meter (CP-505) and a conductivity/oxygen meter (CCO-501), respectively. The total carbon and nitrogen contents in samples were analyzed using a CNS analyzer (Vario MAX Cube, Elementar, Analysensysteme, Langenselbold, Germany). The total content of the elements was assessed by mineralizing the samples in a chamber furnace at 450 °C for 8 h. Redistilled water was used to extract heavy metals from compost (maintaining a material-to-water ratio of 1:10). The elemental content was determined using the ICP-OES technique (ICP-OES Optima 7300DV from Perkin Elmer, Inc., Waltham, MA, USA). The effect of additives in the composting process on the immobilization of metals was examined by calculating the percentage of heavy metals extracted with water in the total content. Metals extracted with water are considered highly mobile and thus easily mobilized, making them readily bioavailable and, as a result, posing a high ecological risk [24]. The Risk Assessment Code (RAC) was applied to evaluate the risk associated with metal release [25].

The humic acid content in composts was determined according to the Schnitzer method [26]. The Cha/Cfa ratio was employed as an indicator of compost stability when greater than 1.6 [27].

### 2.3. Biochemical Analyses in Composts

Enzymatic activity of dehydrogenases was determined by colorimetry using a Backman DU 640 spectrophotometer at a wavelength of 485 nm [28]. The respiratory activity of composts was determined using the manometric method with Oxi-Top measuring equipment, in accordance with ISO 14855-1:2012 [29,30]. Respiratory activity was calculated using the ideal gas equation and estimated to 0.001 mg O_2_·g·h according to the formula given below. The results are presented as functions of 1°, where “y” is the oxygen demand in mg O_2_/g of compost and “x” is the time expressed in hours (h). The lag phase was taken into account, which in the case of this study lasted 4 days (96 h), and was determined based on the start of activity in the PL + BC + SPP treatment.

(1) $$RA=\frac{MO_{2}}{R\cdot T}\cdot \frac{v_{f_{r}}}{mB_{t}}\cdot \left|∆p\right|[mgO_{2}\cdot (g\cdot h{)}^{-1}]$$

where RA—respiratory activity; 

MO_2_—molar mass O_2_ (31.999 g·mol^−1^);

R—ideal gas constant; 83.14 L·hPa·(K·mol)^−1^;

T—temperature (K);

mB_t_—dry matter of material (kg);

|Δp|—pressure changes (hPa);

$v_{f_{r}}$—free gas volume (dm^3^). 

### 2.4. Ecotoxicity Analysis of Composts

The Phytotoxkit and Microtox assays [31,32] were used to ascertain the ecotoxicity of the liquid phase (water extracts). Water extracts were prepared from compost samples (material:distilled water; 1:10). The extraction process was conducted for 24 h. Two plants were used in the Phytotoxkit assay: *Lepidium sativum* and *Sorghum saccharatum*. The assay was conducted in 3 replicates according to the standard procedure [31]. Inhibition of germination (IG%) and root growth (IR%) were determined. The Microtox assay assessed the inhibition of luminescence in *Alivibrio fischeri* bacteria. The procedure was carried out using Screening Test 81.9% and the M 500 Analyzer [32]. In the Ostracodtoxicity test, the crustacean Heterocypris incongruens was used as the test organism. Following a six-day incubation period, the mortality and growth inhibition of *H. incongruens* were measured [33].

The materials were then categorised according to their toxicity class.: Class I (PE ≤ 20%): non-toxic sample; Class II (20% < PE ≤ 50%): low toxic sample; Class III (50% < PE < 100%): toxic sample; Class IV (PE = 100%): highly toxic sample [34].

### 2.5. Statistics

One-way analysis of variance (ANOVA) and Tukey’s test (HSD) were used to analyse the significance of the differences between the mean values of the parameters at a significance level of *p* ≤ 0.05. Pearson’s correlation was performed to identify potential relationships between metal content and the ecotoxicity of the samples. The statistical software Statistica (version 13.1, StatSoft Inc., Tulsa, OK, USA) and Microsoft Excel 2016 were used for the analysis.

## 3. Results and Discussion

### 3.1. The Changes in Temperature During Composting

The physical structure of biomass is a critical factor in the efficacy of the composting process, as it determines the living conditions of the microorganisms involved. One quantifiable effect that substantiates the beneficial properties of composted material for microorganisms is the course of temperature changes [35]. Enriching composted materials with biochar promotes the loosening of biomass during composting and increases the total porosity of compost structures. In our own study, the mixture of PL+BC was the starting material, which was additionally supplemented with γ-polyglutamic acid (PGA), superphosphate (SPP), and smectite-silica clay (SSC) (Figure 1).

It is noteworthy that, with the exception of the initial two weeks of the composting process, during which the incorporation of PGA exhibited the most significant effect on temperature elevation, the subsequent two-week periods saw a consistent temperature in the treatments with the addition of PGA, SPP, and SSC. The lowest temperatures were recorded for composted poultry litter without additives (PL), and the addition of BC only affected the temperature in the first 15 days.

### 3.2. Compost Parameters

The dry matter (DM) content in samples to which PGA, SPP, or SSC was added to PL+BC ranged from 398.6 g to 436.9 g kg^−1^. The lowest DM content was determined in PL+BC+SPP compost, and the highest DM content was found in composted PL without additives (Table 2).

The addition of BC to poultry litter (PL) and supplementation of PL+BC with other additives (PGA, SPP, SSC) reduced the pH values of composts compared to PL (Table 2). However, the pH range of all composts, with the selected substrate proportions, was close to neutral. Significantly the lowest pH value was recorded in BC+PL+SPP compost. The acidifying effect of superphosphate additive to composted sewage sludge and pig manure is confirmed in the studies of Yuan et al. [36] and Yang et al. [37]. The observed acidifying effect of SPP addition to composted biomass is likely attributable to the neutral pH of SPP, which is associated with its chemical composition, specifically the presence of Ca(H_2_PO_4_)_2_ and potentially residual contents of free H_3_PO_4_ and H_2_SO_4_.

The biological transformation of biomass is accompanied by, inter alia, the release of mineral compounds from organic compounds, including NH_4_^+^, NO_3_^−^, PO_4_^3−^, K^+^, Ca^2+^, and Mg^2+^, which can affect electrical conductivity (EC). EC measurement is a reliable indicator of compost salinity, which may determine its natural use. The reduction in EC values compared to PL compost can be regarded as an important effect of the additives. Among the composts produced on the basis of PL and BC, the highest EC value was determined in the compost with the addition of superphosphate (PL+BC+SPP). The EC value determined in the PL+BC+SPP sample was significantly lower than that determined in composted PL without additives. However, it exceeded the recommended standard value of 4 mS cm^−1^ reported in the study by Chen et al. [20]. The rise in EC following the addition of superphosphate to composted biomass has also been observed by other researchers. This phenomenon is likely attributable to the release of PO_4_^3−^, HPO_4_^2−^, H_2_PO_4_^−^, and Ca^2+^ ions from superphosphate, a process that is facilitated by organic acids produced during the thermophilic phase of composting [20,38]. addition, the poultry litter applied in our own study exhibited a comparatively elevated EC value (3.76 mS cm^−1^). In PL+BC, PL+BC+PGA, and BC+PL+SSC composts, the EC values were lower, reaching 7.96 mS, 7.89 mS, and 7.48 mS cm^−1^, respectively. When analyzing the reasons for the increase in EC values in composts, it is also important to point out the effect of compaction resulting from the loss of organic matter as a result of composting. This effect is clearly confirmed by the EC value determined in composted PL without additives (10.53 mS cm^−1^).

The total nitrogen (Nt) content was lowest in composted PL without additives (Table 2). The highest Nt content was recorded for PL+BC (16.14 g kg^−1^ dm). Taking the Nt content in PL+BC compost as a reference point, it should be noted that the addition of PGA, SSC, and SPP did not have a beneficial effect on increasing the content of this component in the composted material, which, especially in the case of SPP, contradicts the results of other researchers [20,21,39]. Given the chemical nature of PGA, its addition into composted biomass is expected to mitigate nitrogen losses. The underlying mechanism of this process is predicated on the sorption of ammonium ions and a concomitant decrease in the pH of the environment. This, in turn, exerts an influence on the course of nitrification, not only in the compost environment but also in the soil [18]. Our study did not confirm the beneficial effect of either SPP or PGA on increasing the Nt content in composts, which was probably due to the dosage of additives used.

### 3.3. Fractional Organic Matter Composition and Bioavailable Content of Heavy Metals of Composts

The addition of BC alone to PL significantly increased the content of this component in the final product due to the high Ct load (Table 3). Enriching PL+BC with PGA, SPP, and SSC additives contributed to an increase (by an average of 6%) in the Ct content in the final product, which can be equated with a reduction in carbon losses during composting.

The content of humic acids (Cfa, Cha) and hemicellulose carbon (Chm) was significantly higher in composted PL. The incorporation of SSC proved to be a substantially more efficacious method of enhancing the Cha content in compost, exhibiting an average increase of 23% compared to the effects of PGA and SPP. This finding is consistent with the results of Shen et al.’s study [38], which showed that incorporating clay minerals into composted biomass significantly increased humic acid content by over 23% compared to control compost. Liquid chromatography with mass spectrometry and spectroscopic analyses conducted by Shen et al. [38] showed a higher degree of aromatization of humic acids in compost with the addition of clay minerals, which proves that their addition promotes mutual conversion between functional groups. The addition of BC and SSC had a significant effect on the higher Chm content in composted PL.

The content of non-hydrolyzable carbon (Cnh)—a relatively stable fraction of biologically transformed organic matter—is important for the stability of composts in the environment. The incorporation of biochar into PL resulted in a substantial increase in this fraction of humic compounds, and the subsequent addition of PGA, SPP, and SSC further stabilized this content, culminating in an average Cnh content increase of over 7%.

The Cha/Cfa ratio is a critical metric for evaluating the stability of organic matter [17]. In addition to composted PL, Cha/Cfa ratio values exceeding 2 were also obtained in PL+BC+SPP and PL+BC+SSC composts, suggesting the presence of enhanced biochemical processes during composting. This hypothesis is further substantiated by the temperature curve observed in both materials during the composting process. The Cha/Cfa ratio of mature compost should be greater than 1.6 [27]. The present experiment indicates that all composts achieved values greater than 1.6. Among the composts to which PGA, SPP, or SSC were added, the highest Cha/Cfa values were obtained after adding SSC (Cha/Cfa ratio 2.15) and SPP (Cha/Cfa ratio 2.11). While the increase in Cha content and stability in the case of SSC addition has been justified [40], the Cha/Cfa ratio in compost with SPP addition was primarily determined by the lower Cfa content.

The incorporation of BC as well as BC and PGA into poultry litter led to a significant (*p* ≤ 0.05) decline in the content of general forms of all the tested elements, reaching the lowest level (see Table 4). The highest content of general forms of the tested metals, except for cadmium, was determined in composted PL without additives, which was the result of thickening. A comparison of the determined contents of general forms of the tested heavy metals with the pollution limits for composted biowaste reveals that, in terms of cadmium and zinc content, composted PL without additives does not meet the set criteria. The addition of superphosphate into compost was also problematic from the point of view of cadmium content [41]. The highest number of cadmium forms extracted with water was found in composted PL without additives (Table 4). However, the highest RAC value was recorded for PL+BC+PGA compost. Significantly, the highest amount of copper forms extracted with water was determined in PL+BC and PL+BC+PGA composts. The determined contents were reflected in relatively high RAC values, which amounted to 31.70% for PL+BC compost and 33.30% for PL+BC+PGA compost. This apparent increase in Cu mobility may seem inconsistent with the overall stabilization effect observed for other metals. However, it should be noted that the behavior of heavy metals during composting is element-specific and strongly influenced by their interactions with organic matter. Copper has a high affinity for dissolved organic compounds, particularly low-molecular-weight fractions formed during composting, which can form soluble complexes and increase its water-extractable fraction. The addition of biochar and PGA may have enhanced the formation of such soluble organic compounds, thereby increasing Cu mobility in PL+BC and PL+BC+PGA treatments. Similar observations have been reported in previous studies, where certain additives promoted the mobilization of Cu while immobilizing other metals. In contrast, the addition of SPP and SSC resulted in lower RAC values, indicating more effective immobilization mechanisms, likely related to phosphate precipitation (SPP) and sorption processes (SSC). A comparable scenario, as in the case of copper, was observed in the content of lead forms extracted with water, albeit with considerably lower RAC values. The determined content of zinc forms extracted with water was highest in composted PL and in PL+BC+PGA compost. Due to the lower overall zinc content in compost with PGA addition, the RAC value was highest for that compost.

This study did not give a clear answer as to the impact of BC and other additives on the immobilisation of the tested heavy metals (HM). When analyzing the content of individual HM in PL+BC composts, it should be noted that the addition of SPP and SSC had a superior effect on the immobilization of these elements than the addition of BC alone or BC and PGA. The reduction in the bioavailability of heavy metals as a result of adding clay minerals or superphosphate to composted poultry litter has been confirmed by other researchers [19,42,43]. However, it is imperative to acknowledge the potential existence of other mechanisms that impose limitations on HM mobility. Although SPP addition lowered the pH of the compost, no excessive mobilization of heavy metals was observed. The reduction in metal bioavailability likely results from a combination of the pH effect and direct interactions with organic matter and phosphates, highlighting that pH-driven changes and immobilization mechanisms act together but remain mechanistically distinct. In the instance of clay minerals, the sorption mechanism may have been dominant due to their large specific surface area and negative charge. In earlier studies, Gondek et al. [22] demonstrated that the incorporation of diatomite into the poultry litter composting process had a substantial impact on the immobilization of cadmium and zinc. In the case of copper, it was observed that each functionalized material (biochar, diatomite, zeolite) contributed to the mobilization of bioavailable forms of this element. The cited authors made analogous observations for lead, except for the compost amended with biochar and bacterial metabolites. These observations suggest that, in addition to introducing various types of additives to the waste composting process, it is important to create specific conditions that will promote the HM immobilization process, such as adjusting the pH or the content of humic compounds rich in functional groups. The chemical behavior of the element in the matrix, which is a composted biomass, is also a contributing factor. Furthermore, the appropriate preparation of the additive, i.e., its functionalization for a specific action (passivating action in relation to HM in this case), may play an important role. It should be noted that only a single additive dose (0.5%) was used in this study, which limits the ability to assess dose–response relationships and determine optimal application rates. Therefore, the observed effects should be considered specific to this concentration.

### 3.4. Dehydrogenase Activity and Respiratory Activity of Composts

**Figure 2:**
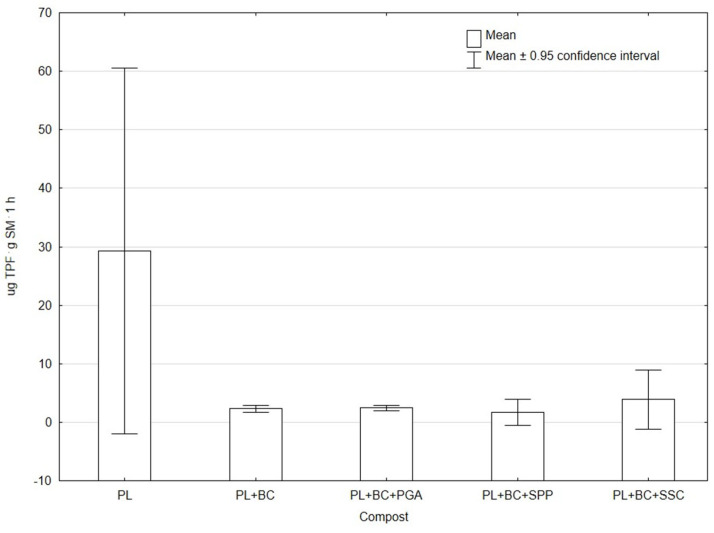
DhA activity in composts.

The measured dehydrogenase activity (DhA) in all composts to which BC was added to PL was lower than the DhA value measured in composted PL without additives (Figure 2).

The incorporation of PGA, SPP, and SSC into the composted PL+BC biomass did not result in any substantial alterations to the DhA value. Our findings suggest that the incorporation of PL with BC and other additives may enhance the biochemical stability of composts. Since the additives used in PL have a beneficial effect on the biochemical processes occurring during biomass composting (improved aeration, moisture stabilization, NH_4_^+^ adsorption, neutralization of toxic substances), it can be assumed that the higher level of DhA activity concerned the earlier stages of composting, as indicated by the results of other researchers [35,44]. Mineral additives for composting can fulfil various functions, such as binding or neutralising pollutants [45,46]. However, their beneficial effect on microbial activity may be limited.

The trend lines for compost respiratory activity (Table 5) during the study period (96 to 312 h of incubation) exhibited a high fit value, as indicated by the coefficient of determination. However, slightly lower values of this coefficient were observed for PL+BC composts with the addition of PGA and SPP, which can be attributed to the lag phase delay. The tested materials, including PL compost, showed minimal biological activity, suggesting that the composting process has led to the formation of stable and mature materials. In the PL treatment, the material demonstrated the most significant biological activity, exhibiting a substantial difference from the other materials. This is evidenced by the parameter “a” of the equation and the cumulative oxygen demand (Table 5). In comparison with other treatments, PL compost demonstrated the most rapid response to the initiation of the incubation process (constant b = 1.5711 mg O_2_ in 96 h). All other combinations involving the addition of biochar or other additives (PGA, SPP, SCC) to PL+BC resulted in a decline in the activity of the compost obtained. The incorporation of biochar and substrates led to an increase in respiratory activity, reaching up to 0.010 mg O_2_·g^−1^·h^−1^. The cumulative respiratory activity of the composts obtained with additives differed significantly (*p* ≤ 0.05) from that of PL compost. The lowest cumulative respiratory activity was observed in compost with the addition of PL+BC+SPP, which corresponds to the dry matter and pH data (Table 3). The respiratory activity of the composts serves to confirm the DhA relationships in these materials (Figure 2). The stabilization of the composts, as indicated by their respiratory activity, is likely attributable to enhanced aeration conditions, primarily resulting from the incorporation of BC into PL during the preparation phase for composting.

### 3.5. Ecotoxicity of Composts

The ecotoxicity of composts varied in their response to test organisms. However, it is noteworthy that, with the exception of the test with *A. fischeri* bacteria, no substantial alterations in ecotoxicity were observed between the examined treatments (Table 6). The greatest inhibition of root growth in test plants was observed in treatments where PGA (*S. alba*) and PGA and SPP (*S. saccharatum*) were added to the composted PL+BC biomass. In turn, the lowest phytotoxicity was found in PL+BC, PL+BC+SPP for *S. alba* and PL+BC for *S. saccharatum*. It is also noteworthy that for *S. alba*, the composts PL, PL+BC, PL+BC+SPP, and for *S. saccharatum*, the composts PL, PL+BC, were non-toxic. The remaining composts exhibited low phytotoxicity (20% < PE ≤ 50%). In the case of *H. incongruens* and *A. fischeri*, the ecotoxicity determined in all composts to which additives were added to PL was lower than the ecotoxicity observed in composted PL without additives (Table 6). The lowest inhibition of *H. incongruens* growth was observed in PL+BC+PGA treatments, and mortality in PL+BC and PL+BC+SSC treatments. The lowest inhibition of *A. fischeri* luminescence was observed in samples with PL+BC+SPP and PL+BC+SSC. Composts in which other materials were added to PL+BC were slightly toxic to *H. incongruens* and *A. fischeri*, while compost from PL alone was toxic to both organisms. When classifying the tested composts based on all test reactions, PL compost was classified as toxicity class III (significant toxicity), while all composts in which BC, PGA, SPP, and SSC were added to PL were classified as toxicity class II (low toxicity). As with dehydrogenases, the reduced ecotoxicity of composts with additives may have resulted from their beneficial effect on the binding or neutralization of pollutants, or the improvement of other physicochemical parameters of the compost. Among the organisms examined, *A. fischeri* exhibited the greatest sensitivity to the analyzed composts, with *H. incongruens* demonstrating a close second. The test plants demonstrated the least sensitivity. The variation in the sensitivity of test organisms may be associated with the method by which the organism is exposed to toxic substances. In the Ostracodtoxkit assay, organisms were exposed to both soluble substances and contaminants adsorbed on solid particles of mixtures. In contrast, the exposure of *A. fischeri* and test plants was related to readily available soluble compounds in water. However, the results obtained do not provide clear evidence as to which additive had the greatest effect on reducing/increasing ecotoxicity. This phenomenon is a recurring theme in research examining the toxicity of diverse waste materials. In bioassays, the response of a living organism is used to assess the overall toxicity of all substances present in the test sample. Often, these substances interact synergistically or antagonistically [25,47,48]. The issue of compost ecotoxicity is of significant importance, as waste composting is increasingly incorporated into waste management strategies in Poland and worldwide [49,50,51]. Furthermore, the utilization of bioassays facilitates the evaluation of potential risks associated with the application of diverse materials for agricultural and reclamation purposes [49,50,52,53,54]. Statistical analysis revealed significant positive correlations between total Cu content and the responses of *A. fischeri* (r = 0.71, *p* < 0.05) and *H. incongruens* (MR = 0.65, *p* < 0.05; LI = 0.67, *p* ≤ 0.05), as well as between total Zn content and sample toxicity to *A. fischeri* (r = 0.76, *p* ≤ 0.05). Significant positive correlations were also found between the content of water-soluble forms of Cd and Zn, and the inhibition of *A. fischeri* luminescence (r = 0.90 and r = 0.73, respectively, *p* ≤ 0.05), as well as between Cd and the mortality of *H. incongruens* (r = 0.67, *p* ≤ 0.05).

The results indicate that the toxicity of the samples to the test organisms is affected by their metal content. Bioassays can be used for ecological risk assessments. However, further research is required in each case to establish the correlation between the presence of heavy metals in the samples and the response of the test organisms. The diversity of responses from test organisms, as well as their relationships with elements or the matrix, can affect the actual ecotoxicity of the samples [25]. The absence of significant relationships between the ecotoxicity of the samples and their heavy metal content can be explained by the fact that biotests provide an overall assessment of the toxicity of all the substances present in the tested sample, many of which act synergistically or antagonistically [52,54].

## 4. Conclusions

The incorporation of PGA, SPP, and SSC into PL+BC composts significantly enhanced organic matter stability and altered heavy metal behavior. All additives increased total carbon (Ct), with SSC being the most effective, raising humic acid carbon (Cha) by an average of 23% compared to PGA and SPP. Biochar addition alone markedly increased non-hydrolyzable carbon (Cnh), while the inclusion of PGA, SPP, or SSC further elevated Cnh by an average of 7%, indicating improved humification and compost maturity. All composts achieved Cha/Cfa ratios above 1.6, with the highest values observed for PL+BC+SSC and PL+BC+SPP; however, the increase in the SPP variant primarily reflected a reduction in fulvic acids carbon (Cfa) rather than enhanced humification. Heavy metal analysis revealed that SPP addition may raise cadmium levels, likely due to contaminants in the raw fertilizer material, whereas water-extractable Cd, Cu, Pb, and Zn were lowest in PL+BC+SPP, as reflected in reduced RAC values. Overall, additive-enriched composts achieved lower ecotoxicity (toxicity class II) compared to unamended PL (class III), with the greatest sensitivity observed in A. fischeri and H. incongruens, and minimal phytotoxic effects on test plants. These effects are likely due to improved binding or neutralization of pollutants and enhanced physicochemical properties. The combined analysis of carbon stabilization, metal immobilization, ecotoxicity, and composting dynamics demonstrates that additive enrichment, particularly with SSC and SPP, effectively enhances compost quality. The study highlights the potential of carefully selected additives to accelerate biological stabilization, reduce toxicity, and improve the agronomic value of poultry litter–biochar composts. Future research should explore a wider range of additive doses to optimize their application and evaluate dose-dependent effects on organic matter transformation, metal bioavailability, and ecotoxicity.

## Figures and Tables

**Table 1:** Selected chemical parameters of materials used for composting (mean ± SD).

Material		PL ^1^	BC ^2^	SPP ^3^	SSC ^4^
Dry matter	g kg^−1^	862 ± 10	948 ± 11	990 ± 1	965 ± 1
Nt ^5^	g kg^−1^ dm	27.29 ± 1.14	6.86 ± 0.38	-	0.89 ± 0.04
Ct ^6^		280 ± 5	444 ± 3	-	1.9 ± 0.2
pH		6.63 ± 0.09	7.52 ± 0.03	3.30 ± 0.04	6.82 ± 0.13
EC ^7^	mS cm^−1^	3.76 ± 0.10	0.22 ± 0.03	8.40 ± 0.10	0.06 ± 0.04
Cd	mg kg^−1^ dm	0.74 ± 0.04	0.29 ± 0.01	13.87 ± 0.23	0.13 ± 0.02
Cu		18.38 ± 0.32	9.76 ± 0.50	14.85 ± 0.04	68.13 ±4.40
Pb		7.88 ± 1.66	1.55 ± 0.01	2.82 ± 0.85	7.10 ±0.49
Zn		197.8 ± 0.9	88.1 ± 8.1	166.6 ± 12.8	43.7 ± 0.8

^1^ PL—poultry litter, ^2^ BC—biochar, ^3^ SPP—superphosphate, ^4^ SSC—smectite-siliceous clay, ^5^ Nt—total nitrogen, ^6^ Ct—total carbon, ^7^ EC—electrolytic conductivity.

**Table 2:** Basic properties of compost.

Compost	Dry Matter	pH	EC ^1^	Nt ^2^
	g kg^−1^		mS cm^−1^	g kg^−1^ dm
PL	573.4 ± 0.1 c ^3^	6.96 ± 0.01 b	10.53 ± 1.24 b	14.47 ± 0.42 a
PL+BC	442.7 ± 0.2 b	6.68 ± 0.02 b	7.96 ± 0.01 a	16.14 ± 0.38 a
PL+BC+PGA	424.8 ± 0.1 b	6.70 ± 0.02 b	7.89 ± 0.62 a	15.40 ± 0.76 a
PL+BC+SPP	398.6 ± 0.1 a	6.19 ± 0.01 a	8.77 ± 0.20 ab	15.85 ± 0.02 a
PL+BC+SSC	436.9 ± 0.2 b	6.66 ± 0.04 b	7.48 ± 0.17 a	15.18 ± 0.05 a

^1^ EC—electrolitical conductivity, ^2^ Nt—total nitrogen, ^3^ The same letters in the columns indicate that there are no significant differences between the values at the level of significance (*p* ≤ 0.05) according to Tukey’s HSD tests.

**Table 3:** Fractional composition of humic compounds in composts (Mean ± SD).

Compost	Ct ^1^	Cfa ^2^	Cha ^3^	Chm ^4^	Cnh ^5^	Cha/Cfa Ratio
	**g kg^−1^ dm**					
PL	115.1 ± 3.9 a ^6^	14.71 ± 0.16 b	32.64 ± 1.53 b	13.72 ± 0.45 c	54.0 ± 1.3 a	2.22 ± 0.03 b
PL+BC	285.3 ± 3.2 b	7.93 ± 0.35 a	13.61 ± 3.33 a	8.13 ± 0.74 b	255.6 ± 2.9 b	1.72 ± 0.25 a
PL+BC+PGA	301.9 ± 3.1 c	7.66 ± 0.27 a	12.71 ± 1.02 a	6.58 ± 0.18 a	275.0 ± 3.1 b	1.66 ± 0.06 a
PL+BC+SPP	303.3 ± 0.8 c	6.05 ± 0.57 a	12.79 ± 1.19 a	6.36 ± 0.16 a	278.4 ± 0.8 b	2.11 ± 0.10 b
PL+BC+SSC	302.9 ± 1.5 c	7.29 ± 0.89 a	15.70 ± 1.42 a	8.05 ± 0.19 b	271.9 ± 1.4 b	2.15 ± 0.11 b

^1^ Ct—total carbon, ^2^ Cfa—fulvic acids carbon, ^3^ Cha—humic acids carbon, ^4^ Chm—hemicellulose carbon, ^5^ Cnh—nonhydrolizing carbon, ^6^ The same letters in the columns indicate that there are no significant differences between the values at the level of significance (*p* ≤ 0.05) according to Tukey’s HSD tests.

**Table 4:** The total and bioavailable content of HM and the RAC index value in composts (mean ± SD).

Compost	Cd	Cu	Pb	Zn
	Total Forms, mg kg^−1^ dm			
PL	0.808 ± 0.000 b ^2^	22.15 ± 0.221 b	8.87 ± 0.06 a	211.5 ± 6.8 b
PL+BC	0.563 ± 0.006 a	15.20 ± 0.54 a	5.42 ± 0.68 a	157.4 ± 3.1 a
PL+BC+PGA	0.525 ± 0.011 a	15.00 ± 0.76 a	5.77 ± 1.55 a	155.8 ± 4.8 a
PL+BC+SPP	0.822 ± 0.060 b	18.94 ± 0.38 ab	8.06 ± 1.30 a	181.2 ± 1.5 ab
PL+BC+SSC	0.630 ± 0.034 a	16.54 ± 0.40 ab	6.63 ± 0.03 a	169.1 ± 3.8 ab
Compost	H_2_O extraction, mg kg^−1^ dm			
PL	0.083 ± 0.006 c	3.06 ± 0.04 a	0.183 ± 0.020 a	6.07 ± 0.08 c
PL+BC	0.047 ± 0.002 ab	4.81 ± 0.21 c	0.200 ± 0.017 a	5.12 ± 0.08 b
PL+BC+PGA	0.064 ± 0.008 bc	4.99 ± 0.08 c	0.194 ± 0.021 a	5.77 ± 0.22 bc
PL+BC+SPP	0.030 ± 0.001 a	3.60 ± 0.39 ab	0.159 ± 0.016 a	3.71 ± 0.39 a
PL+BC+SSC	0.035 ± 0.003 a	3.95 ± 0.09 b	0.165 ± 0.005 a	4.17 ± 0.09 a
Compost	RAC ^1^ %			
PL	10.27 cd	13.81 a	2.06 a	2.87 ab
PL+BC	8.28 bc	31.70 c	3.70 c	3.25 ab
PL+BC+PGA	12.24 d	33.30 c	3.44 bc	3.73 b
PL+BC+SPP	3.61 a	19.11 ab	1.99 a	2.08 a
PL+BC+SSC	5.93 ab	23.87 b	2.49 ab	2.46 a
Contamination limits, mg kg^−1^ dm				
composted or digested bio-waste ^3^	0.7	70	45	200

^1^ RAC—Risk Assessment Code, ^2^ The same letter in columns means no significant differences between the values at the level of significance (*p* ≤ 0.05) according to Tukey’s HSD tests, ^3^ Walk and Gambini [41].

**Table 5:** Trend lines of changes in respiratory activity and cumulative oxygen demand of poultry-litter-based composts.

Treatment	Equation of the 1° Activity Curve (RA) During the Incubation Period of 96–312 h, [mg O_2_·g^−1^ dm]		Cumulative Oxygen Demand in 312 h, [mg O_2_·g^−1^ dm]
PL	^1^ y = 0.0214x + 1.5711	R^2^ = 0.9988	6.202 b ^2^
PL+BC	y = 0.0080x + 0.2187	R^2^ = 0.9760	1.995 a
PL+BC+PGA	y = 0.0072x + 0.5641	R^2^ = 0.9400	2.172 a
PL+BC+SPP	y = 0.0071x + 0.0242	R^2^ = 0.9461	1.564 a
PL+BC+SCC	y = 0.0092x + 0.2988	R^2^ = 0.9782	2.186 a

^1^ y = ax + b (x—hour), ^2^ The same letter in columns means no significant differences between the values at the level of significance (*p* ≤ 0.05) according to Tukey’s HSD tests.

**Table 6:** Ecotoxicity of composts (mean ± SD).

Compost	^2^ RGI%		^3^ M%	^4^ LI %	^5^ LI %	Class Toxicity
	*S. alba*	*S. saccharatum*	*H. incongruens*		*A. fischeri*	
PL	10 ± 13 a ^1^	13 ± 4 a	40 ± 5 a	51 ± 1 a	72 ± 1 b	III
PL+BC	5 ± 7 a	7 ± 3 a	0 ± 0 a	39 ± 1 a	50 ± 1 a	II
PL+BC+PGA	27 ± 15 a	23 ± 12 a	15 ± 5 a	31 ± 8 a	49 ± 1 a	II
PL+BC+SPP	4 ± 8 a	26 ± 3 a	20 ± 5 a	36 ± 8 a	46 ± 3 a	II
PL+BC+SSC	18 ± 3 a	21 ± 9 a	0 ± 0 a	41 ± 2 a	46 ± 2 a	II

^1^ The same letter in columns means no significant differences between the values at the level of significance (*p* ≤ 0.05) according to Tukey’s HSD tests, ^2^ Roots growth inhibition (Phytotoxkit), ^3^ mortality (Ostracodtoxkit), ^4^ length inhibition (Ostracodtoxkit), ^5^ luminescence inhibition (Microtox).

## Data Availability

The original contributions presented in this study are included in the article. Further inquiries can be directed to the corresponding author.
